# THE VOICE OF OLDER ADULTS ON DIGITAL INCLUSION FOR HEALTHY COMMUNITY IN SINGAPORE

**DOI:** 10.1093/geroni/igad104.0741

**Published:** 2023-12-21

**Authors:** Carol Ma

**Affiliations:** Singapore University of Social Sciences, Singapore, Singapore

## Abstract

With the rapid ageing population and the experience of Covid-19 pandemic, there is an urgent need to create a digitally inclusive healthy society. Singapore, as a smart nation, has been at the forefront of promoting digital inclusion to all age groups, including older adults. According to the Statista (2022), 88.5% of the population in Singapore are using internet and 56% of those over 60 are smartphone users. The use of digital technology for health promotion holds enormous potential to empower individuals and communities, particularly in the context of healthy and active ageing. The older adults in this study are adept at using the basic function of the smart phone rather than a computer. Some have even attended the national program, “Seniors Go Digital,” to learn about basic communication tools, file messaging, video calling, accessing government digital services, using e-payment tools, etc. They have also shared the barriers they face in using the internet, such as declines in vision, cognitive ability, and hearing, as well as the opportunities they have found, such as arrange telemedicine and access to the e-health systems. Besides, they found that the peer support and social networks are very important to support their digital learning journey. Though fear of encountering cheats and scams is common, they can be overcome by building confidence through peer support and self-efficacy. During the presentation, the authors will identify the contextual influences of digital inclusion and its impacts on healthy ageing in Singapore.

